# A Systematic Review of the Long-Term Trajectory of Hemodynamics and Body Composition in Childhood Obesity

**DOI:** 10.7759/cureus.19504

**Published:** 2021-11-12

**Authors:** Megha Suri, Anuj Suri, Deepali Kumar, Rohini Patel

**Affiliations:** 1 Medicine-Pediatrics, California Institute of Behavioral Neurosciences & Psychology, Fairfield, USA; 2 Internal Medicine, St. Michael's Hospital, Toronto, CAN; 3 Internal Medicine, Windsor University School of Medicine, Chicago, USA

**Keywords:** obesity adolescent anthropometry, hemodynamics, diastolic blood pressure, cardiovascular risks, hypertension in young patients, body mass index: bmi, childhood obesity

## Abstract

Obesity has long been cited as a significant risk factor for high blood pressure, with long-term exposure linked with an increased incidence of carotid artery atherosclerosis. However, as obesity is becoming more common in young-aged children, it is important to recognize combinational factors like lifestyle, socioeconomic, and genetic factors as a cause. Increasing weight during childhood, on the other hand, is a clinically significant challenge for many physicians. It is critical to identify these risk factors since early prevention (primordial prevention) or treatment (primary prevention) to reverse the potential risks is much more likely to be effective. The objective of this review was to explore the relationship between lifestyle, anthropometric, and genetic factors and cardiometabolic risk factors. We are hopeful that our findings may aid in the primary prevention of hypertension in children and the prevention of any related cardiovascular complications that may arise later in life as a result of obesity. The synthesis of this systematic review includes a total of 15 studies using defined criteria, published up to October 2021. In addition, a total of 2,397 articles were found through an initial electronic database search and included a total of 38,182 participants. Studies explored included one or more of the following cardiovascular risk factors: body mass index (BMI), systolic blood pressure (SBP), diastolic blood pressure (DBP), high-density lipoprotein (HDL) cholesterol, low-density lipoprotein (LDL) cholesterol, and triglycerides (TG). The findings of this study support the notion that childhood obesity may be a risk factor for a variety of adult cardiovascular disease risk factors. Several behavioral, genetic, and anthropometric factors are linked to the development of obesity during early ages, overall creating challenges in cardiovascular health during adulthood. As a result, addressing the risk factors for childhood hypertension would be advantageous for the primary prevention of its sequelae in adulthood.

## Introduction and background

Cardiovascular diseases are part of a well-known reason for death among many individuals. They are responsible for over 17.5 million deaths worldwide and account for 31% of the overall death rate. The estimated number of deaths due to heart disease is forecasted to increase to 23.6 million per year by 2030 [1]. Obesity is one of the known conditions likely to increase the risk of cardiovascular death. Evidence suggests that obese subjects are almost twice as likely to develop cardiovascular disease [2]. Some studies have also shown that childhood obesity is a strong indicator for future health problems in adulthood. However, the extent to which obesity is responsible for cardiovascular disease depends mainly on metabolic cardiovascular risk factors [3]. Some of the included associations found in children include higher blood pressure (BP), impaired glucose tolerance, a poor lipid profile including higher low-density lipoprotein (LDL), triglycerides (TG), and lower high-density lipoprotein (HDL) cholesterol [4-7]. Interestingly, a population-based study found that between the ages of 5 and 17 years, about 70% of overweight children and adolescents present with at least one risk factor for cardiovascular disease [8].

The high prevalence of hypertension in children is a significant concern and a therapeutic challenge for many healthcare providers. Thus, healthcare providers should focus on factors associated with childhood hypertension to prevent the later complications of this disease. This review aimed to assess the association between patterns of lifestyle, anthropometric, and genetic factors and cardio-metabolic risk factors. We intend that our results will be helpful for the primary prevention of hypertension in children and prevent any related complications that may occur later in adulthood due to obesity.

## Review

Methods

The results of this systematic review are reported using the Preferred Reporting Items for Systematic Reviews and Meta-Analyses (PRISMA) guidelines [9].

Search Strategy

A literature review on this topic was conducted from August 6, 2021 to October 30, 2021. Eligible publications were thoroughly reviewed and discovered using a search of PubMed and PubMed Central. To properly filter relevant publications demonstrating childhood obesity, the search technique and Medical Subject Heading (MeSH) phrases and keywords were used. The keywords used include hemodynamics, body composition, blood pressure (BP), systolic blood pressure (SBP), diastolic blood pressure (DBP), and child or children. The Boolean method was applied to the keywords and the MeSH approach format to screen articles in PubMed. Original studies on human subjects published in English were included in the electronic search. Two writers searched for additional citations and retrieved data from each eligible paper independently.

Inclusion and Exclusion Criteria

Two reviewers separately executed the screening process to identify all potentially acceptable citations. Our review included related articles targeting children under the age of 18 years. We limited our study selection to cohort studies, systematic reviews, and meta-analyses of published abstracts and full texts. We excluded studies that did not include author names. Animal studies were also excluded. For final acceptability, papers published in the English language in the last five years, from 2016 and 2021, have been included in the development of this systematic review.

Data Extraction

Data were carefully selected and extracted independently by two researchers (MS and AS) using a standardized recording tool. Characteristics like author names, study design, year of publication, country of study, number of study participants, age, and the study findings were documented.

Methodological Quality Assessment

The Newcastle-Ottawa scale was used to assess the quality of cohort studies, while the Assessment of Multiple Systematic Reviews (AMSTAR) was used to assess the quality of systematic reviews and meta-analyses. Each study was assessed independently using particularized criteria and factors to identify any areas of potential bias. Using this method, we were able to determine the intrinsic methodological quality of each research publication, with scores higher than 8 indicating the point for inclusion.

Results

**Figure 1:**
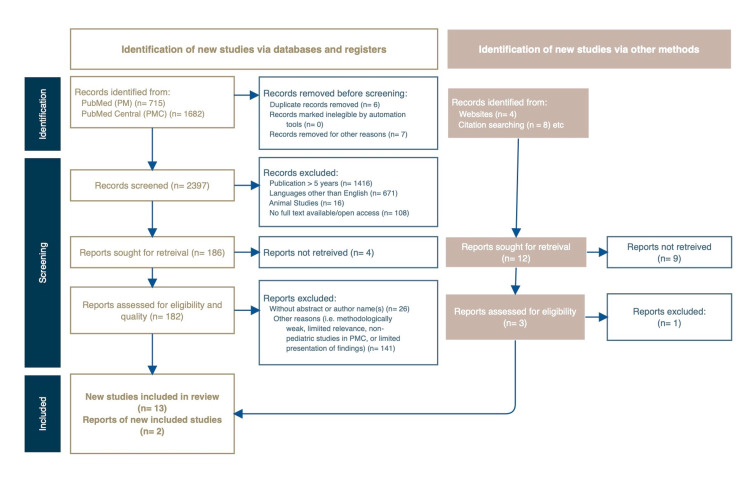
PRISMA flow diagram. PRISMA, Preferred Reporting Items for Systematic Reviews and Meta-Analyses.

Our preliminary search resulted in a total of 2,397 articles. Among the 2,397 articles discovered, 715 were from PubMed, 1,682 studies were found in PubMed Central, and 12 papers were obtained via reference review. Of the total value, we excluded six articles by screening for duplicates and additionally removed 2,211 of them after screening for studies based on their eligibility to our inclusion criteria, matching for the age of participants, years of publication, studies performed in humans, availability of full or open texts, and those published in the English language. The remainder of 186 articles were then filtered based on their respective titles, including those relevant to childhood obesity and adult-related cardiovascular risk factors. The final screening process included 182 articles, in which 167 were discarded due to the lack of results, methodologically weak studies, or reporting limited presentation and findings to our ongoing research. Overall, 15 articles were considered eligible for final analysis. Figure 1 illustrates the performed search according to the Preferred Reporting Items for Systematic Reviews and Meta-Analyses (PRISMA) below.

This article's studies were all unique. However, the intentions were comparable. Table 1 provides a systematic summary of the evaluated literature.

**Table 1:** A tabulated summary of the study characteristics. ACE: angiotensin-converting enzyme; BF: body fat; BP: blood pressure; CVD: cardiovascular disease; DBP: diastolic blood pressure; HDL: high-density lipoprotein; LDL: low-density lipoprotein; OR: odds ratio; PA: physical activity; r: correlation coefficient; RCT: randomized controlled trial; SBP: systolic blood pressure; SD: standard deviation; TG: triglycerides; WC: waist circumference; VLDL: very low-density lipoprotein.

Author	Year	Study design	Journal	Location	Age of participants	Sample size	Follow-up in years	Results
Stoner et al. [10]	2017	Cross-sectional	J Am Heart Assoc	New Zealand	8-10 years old	392	N/A	Researchers discovered that overweight-obese children had higher, and worse risk scores for adiposity, vascular, and cumulative risk scores. Factors identified include BP, cholesterol, adiposity, and vascular factors, of which BP accounted for the vast majority of the heterogeneity.
Umer et al. [11]	2017	Systematic review and meta-analysis	BMC Public Health	United States	N/A	N/A	N/A	According to the findings of the research, childhood obesity may be a risk factor for some adult CVD risk variables. Results outlined that childhood obesity has a positive and significant correlation with developing higher SBP, DBP, and TG during adulthood. In addition, childhood obesity was related to lower adult HDL.
Wade et al. [12]	2017	Cohort	Am J Clin Nutr	United Kingdom	Birth-16 years old	13,557	16 years	Problematic eating patterns in adolescence (11.5 years old) appear to be correlated to the development of obesity. Findings are supported by positive associations between poor eating habits and obesity (OR: 2.18), new-onset high SBP (OR: 1.34), and new-onset high DBP (OR: 1.25) at the age of 16 years. This is a relatively new finding with considerable public health implications regarding obesity control (clinicaltrials.gov: NCT01561612).
Bell et al. [13]	2018	Cohort	J Am Coll Cardiol	United Kingdom	10-18 years old	2,840	8 years	Higher total fat mass index and BMI at 10 years were correlated with higher systolic and diastolic blood pressure, higher VLDL and LDL cholesterol, lower HDL cholesterol, higher TG, and higher insulin and glycoprotein acetyls at 18 years.
Dencker et al. [14]	2018	Cross-sectional	Eur J Pediatr	Sweden	8-11 years old	170	2 years	Higher levels of galectin-3 were associated with higher quantities of body fat and abdominal fat, larger abdominal body fat distribution, greater left ventricular mass, and larger left atrial size. Over a two-year period, an increase in total body fat was directly associated with greater levels of galectin-3.
Sun et al. [15]	2018	Cohort	J Hypertens	Australia	N/A	4,835	23.6 years	The effect of adult obesity on rising blood pressure and the risk of hypertension in adulthood may be modified by ACE genetic polymorphism. Individuals with the ACE DD (or GG) [adjusted linear regression coefficients 0.26, 95% CI (0.21–0.31) and 0.28 (0.24–0.32) for SBP and DBP] and/or ID (or AG) genotypes [0.25 (0.21–0.29) and 0.25 (0.21–0.28)] appear to be more sensitive to the effects of excess adiposity than those with the II (or AA) genotype [0.15 (0.09–0.21) and 0.19 (0.13–0.23)].
Wade et al. [16]	2018	Cohort	Circulation	United Kingdom	17-21 years old	418	3 years	Even in children, higher BMI is associated with lower cardiovascular health, notably higher blood pressure and left ventricular mass index. A higher BMI was also associated with an increase in cardiac output.
Delgado-Floody et al. [17]	2019	Cross-sectional	Nutr Hosp	Chile	Mean age: 12 ± 1.23 years	605	N/A	According to the findings of this study, physical fitness has an inverse association with SBP and a positive link with PA levels. Furthermore, obese children had lower physical fitness and a higher proportion of hypertensive individuals (p < 0.001).
Callo Quinte et al. [2]	2019	Cohort	BMC Pediatr	Brazil	Mean age 30.2 years old	2,219	30 years	The findings suggest that persistent overweight/obesity is related to a worse cardiometabolic profile and that this association is mostly mediated by fat accumulation in adulthood. The impact can be reversed if early intervention to reduce obesity is implemented and thus may overall reduce cardiometabolic risk factors.
Teo et al. [18]	2019	Cohort	PLoS One	Canada	Newborn	761	5 years	At five years old, children in the 90th percentile for BF% or WC had greater levels of TG, glucose, SBP, and DBP than those in the 90th percentile. Such distinctions were not apparent at three years of age or at birth. Additionally, a BMI z-score with more than 2 SD was associated with increased levels of TG, SBP, and DBP, but not glucose.
Angoorani et al. [19]	2020	Cross-sectional	BMC Cardiovasc Disord	Iran	7-18 years old (mean age 12.3 ± 3.1 years)	7,235	N/A	Results of this study strengthen the importance of weight and waist circumference in blood pressure control. Research supports the need to improve dietary habits and health-related behaviors especially in families with low socioeconomic positions. Data: age (r = 0.35 and 0.26, respectively), BMI (r = 0.06 and 0.04, respectively), and WC (r = 0.05 and 0.03, respectively) were all found to be positively associated with systolic and diastolic blood pressure.
Dooley et al. [20]	2020	Cross-sectional RCT	PLoS One	United States	2-12 years old	131	1 year	Light intensity, moderate to vigorous intensity, and total physical activity were linked to favorable decreases in adiposity markers.
Fan et al. [21]	2020	Cohort	J Clin Hypertens	China	2-18 years old	1,444	10 years	Between infancy and adulthood, the prevalence of overweight (including obese) individuals as measured by BMI was 5.7% and 13.4%, respectively. In adulthood, the prevalence of hypertension was 12.7% and 32.9%, respectively. This study investigates the effect of childhood obesity parameters on adult hypertension. Considering sex and childhood age adjustments, child BMI (p < 0.002) and WC (p < 0.001) were strongly linked with adult hypertension.
Norris et al. [22]	2020	Cohort	Arterioscler Thromb Vasc Biol	United Kingdom	7.5-24.5 years old	3,549	24 years	Those with a high and consistent BMI throughout childhood may be at a lower risk of cardiometabolic disease compared to individuals who become overweight or obese in late adolescence. The average BMI trajectory for the "normal weight increasing to obesity" class was substantially greater during early adulthood than the "overweight or obese" class, with evidence of lower levels of numerous health variables.
Czogała et al. [23]	2021	Cross-sectional	Nutrients	Poland	6.6-17.7 years old	26	N/A	By examination of exon 1 of the PLAG1 gene, region 8:56211059–56211208 (genome reference sequence: GRCh38.p13): An increase in PLAG1 expression was linked to a higher BF percentage. The FTO gene is considered to be involved in the development of obesity in children, as well as the coexisting dysfunction of glucose-lipid metabolism.

Discussion

Obesity has been linked to a number of factors that raise the overall risk of acquiring cardiovascular disease during adulthood. Obesity has been also associated with a number of characteristics in children, including increased blood pressure (SBP and DBP), BMI, and waist circumference (WC) in pediatric patients. In light of this huge concern, other considerations on the rise include genetically inherited genes and barriers towards sustaining an active lifestyle. We will go through each of these characteristics individually to shed light on elements that may aid in the overall prevention of issues associated with childhood obesity.

Lifestyle and Anthropometric Factors

With the global epidemic of childhood obesity, high blood pressure has become increasingly common, with a prevalence of at least 5% occurring in children and adolescents [24]. Obesity has been long-recognized as a critical risk factor for high blood pressure, and more recent studies suggest that long-term exposure to high levels is closely associated with an increased risk of early atherosclerosis. Recent research indicates that, as compared to general obesity, central obesity has a more vital link with blood pressure and other cardiovascular risk factors. Blood pressure is affected by blood flow and vascular resistance, influenced by vasoconstriction and stiffness [25]. Furthermore, research shows that blood flow patterns in adolescents are regulated by weight and body composition. High vascular resistance is a primary cause of hypertension and hemodynamic stress on the heart and arteries in the obese, and it is also regarded as an important cardiovascular risk factor [19].

In a study by Angoorani et al., factors like increasing BMI (r = 0.06 and 0.04), WC (r = 0.05 and 0.03), and age (r = 0.35 and 0.26) have been shown to positively correlate with the increase in SBP and DBP [19]. Likewise, a cohort study completed in China following 1,444 children for 10 years aged 4-17 years suggests that childhood BMI and WC are significantly associated with adult hypertension (p < 0.002 and p < 0.001, respectively) [21]. A systematic review and meta-analysis by Umer et al. found a significant and positive association supported by a 95% confidence interval (not including null) between childhood obesity and adult SBP plus DBP, further strengthening the importance of weight and WC in blood pressure control [13]. A cross-sectional study by Stoner et al. published in the *American Heart Association* included 392 children from eight to 18 years old from New Zealand. The study's results propose that overweight-obese children were more likely to experience worse risk scores for adiposity, vascular, and cumulative risk scores due to long-term exposure to high blood pressure [10].

Interestingly, many longitudinal studies also suggest that obesity in childhood is associated with various cardiometabolic risk factors, and the risk may be imposed as early as five years old. Some risk factors noted in the cohort study by Teo et al. of 761 Canadian newborns followed over five years included dyslipidemia, insulin resistance, dysglycemia, and type-2 diabetes mellitus. The strong association found between the incidence of obesity and glycemic control, systolic blood pressures, and lipid levels suggests that atheromatous processes could perhaps begin as early as childhood. Cardiometabolic risk factors can advance the atherosclerotic process and increase the risk of cardiovascular events, especially when prevention and intervention measures are deficient [18]. In a study following 2,219 participants for 30 years, Callo Quinte et al. found that participants who had not been obese or overweight had lower cardiovascular risk factors (low LDL cholesterol and TG, and higher HDL cholesterol). In comparison to participants who were overweight or obese in adulthood, participants had higher cardiovascular risk factors, including the lowest levels of HDL cholesterol and highest levels of LDL cholesterol and triglycerides [2]. Further, obesity is also associated with an increased risk of early death and mortality in adulthood.

**Figure 2:**
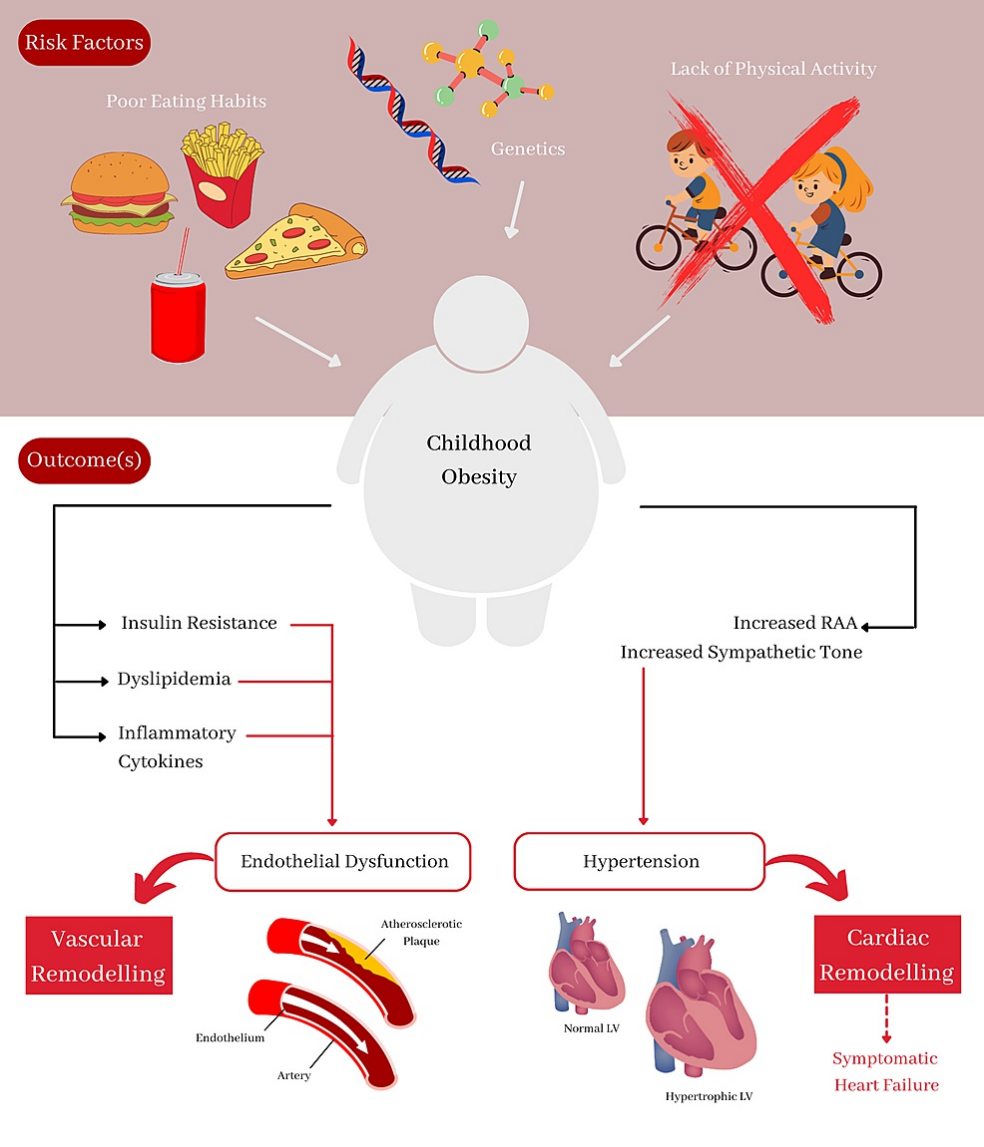
Risk factors and cardiovascular health outcomes related to childhood obesity. RAA: renin-angiotensin-aldosterone; LV: left ventricular.

In correlation with the above results of studies included, uncontrolled eating habits [2,12,19] and lack of physical activity [17,20] are commonly associated findings to the prevalence of childhood obesity. A more recently published cohort by Wade et al. tracks 13,557 newborn pediatric patients from the United Kingdom for over 16 years with observations that problematic eating attitudes in mid-childhood, or around the age of 11.5, can be related to the development of obesity in adolescence [12]. Compared to the cohort of 2018 by Wade et al., studying youth patients aged 17-21 for over five years, the authors analyzed that higher BMI is likely to cause worse cardiovascular health, specifically regarding high BP and a higher left ventricular mass [16]. According to the findings by Bell et al., higher fat mass index and BMI are associated with higher blood pressure, higher very low-density lipoprotein (VLDL) and LDL cholesterol, lower HDL cholesterol, higher triglycerides, and higher glycemic and inflammatory traits, as well as clinical trait precursors such as branched-chain and aromatic amino acids. Overall, the findings confirm the role of abdominal fat as a critical cause of cardio-metabolic dysfunction and BMI as a helpful metric for detecting its consequences [13]. However, another longitudinal study over 24 years by Norris et al. suggests that individuals presenting with a high BMI from childhood to adulthood may have a lower risk of developing certain diseases than subjects who gain most of their weight in late adolescence [22]. Overall, results from various cohort and cross-sectional studies performed globally show that obesity is a significant public health cause of early cardiovascular disease, and regulated implications for obesity reduction are urgently needed to prevent poor health outcomes. Figure 2 illustrates a snapshot of childhood obesity risk factors and outcomes.

Biomarkers for childhood obesity

FTO Expression and Childhood Obesity

In light of the continuing importance of this issue, the role of epigenetics in childhood obesity is becoming a hotly debated topic. Both hereditary and epigenetic factors influence childhood obesity. In a cross-sectional study conducted by Czogała et al., six obese (aged 6.6-17.7 years) and 10 healthy (aged 11.4-16.9 years) children were followed to analyze the expression of the FTO gene (FTO alpha-ketoglutarate dependent dioxygenase), and protooncogene PLAG1 (PLAG1 zinc finger) in the development of childhood obesity [23]. Because these genes regulate fat tissue cell metabolism and are strongly related to body weight, the goal of this study was to examine the genome expression and methylation of the FTO and PLAG1 genes in overweight and obese children. In addition, researchers looked at the effect of FTO gene methylation and both gene expression on anthropometric factors like BMI and WC and biochemical parameters like cholesterol, triglycerides, free fatty acids, and glucose levels.

The FTO gene was considerably more expressed in obese children and patients with established insulin resistance (p = 0.008). Key anthropometric and metabolic variables were associated with FTO methylation and gene expression in both investigated genes. Higher FTO expression was also linked to an increase in WC (cm), body fat (kg and percentile), and a higher fasting free fatty acid (FFA) concentration. Increased PLAG1 expression was linked to a higher body fat percentage, affecting total body composition than the healthy group (p = 0.036). The findings suggest that the FTO gene expression is likely to play an essential role in developing childhood obesity, together with coexisting glucose-lipid metabolism dysfunction.

Overall, this study argues that the FTO gene could play a significant role in developing obesity and the pathophysiology of obesity-related health complications, albeit more research, ideally of a prospective design, is needed to support these findings. As a result of the established positive association between FTO gene synthesis and body fat composition, the increase in BMI observed in the context of FTO expression is caused chiefly by the buildup of adipose tissue rather than lean body mass. Furthermore, a higher FTO expression is independently related to the incidence of insulin resistance. Although environmental variables such as sedentary lifestyle and excess nutrition supply have a significant role in the ultimate result, more emphasis should be on other pertinent obesity-related issues.

Galectin-3 Expression and Childhood Obesity

Galectin-3 is a protein that has been linked to increased collagen synthesis and hypertrophy in response to damage via various pathways. As a novel molecule researched for its regulatory functions within the immune system, galectin-3 levels are associated with clinical consequences in patients with atherothrombosis. Moreover, higher levels of galactin-3 have been shown to correlate with traditional risk factors for cardiovascular diseases associated with mortality in individuals of the general population. Because obese children and adolescents are more likely to acquire adult obesity and have significant short- and long-term health problems [26], it is important to investigate possible associations between galectin-3 versus body fat and other biomarkers for health at a young age. The focus of this research completed by Dencker et al. was to look at the associations between galectin-3 levels and body composition, total body fat, abdominal fat, fat distribution, blood pressure, left ventricular mass, and left atrial size in a community sample of children over two years [14].

A cross-sectional study, including 170 children aged 8-11 years, analyzed participants via dual-energy X-ray absorptiometry (DXA) for total fat mass and abdominal fat. The findings included consistent relationships among galectin-3 levels and all components of body fat measurements (body mass, BMI, total body fat, percent body fat, abdominal fat, body fat distribution, SBP, DBP, pulse pressure, left ventricular mass, left atrial diameter, and relative wall thickness). A strongly associated p-value supported each finding from the study (p = <0.001), suggesting that galactin-3 may very well be a predicting factor bridging childhood obesity and poor cardiovascular risk factor outcomes. However, because of the deficit in data on this topic among children, more research and prospective studies are necessitated to confirm the association between these variables.

ACE Polymorphism and Childhood Obesity

It is estimated that obesity is directly responsible for 60-70% of adult hypertension [27]. As mentioned previously, epigenetic are among some critical risk factors which link childhood obesity to hypertension and cardiovascular disease in adulthood. Researchers were able to investigate the interplay between angiotensin-converting enzyme (ACE) genotypes and childhood obesity for the development of adult high blood pressure and hypertension across three cross-prospective cohort studies, including 4,835 participants. The study discussed here by Sun et al. identified the effect of adult obesity on rising blood pressure and the risk of hypertension in adulthood might be modified by ACE genetic polymorphism [15]. Their research demonstrates that the ACE DD genotype has a more significant and negative influence on various cardio-metabolic outcomes, including triglycerides and insulin resistance than other ACE I/D genotype carriers. The findings also included individuals with the ACE DD (or GG) and ID (or AG) genotypes, suggesting they are likely to be more sensitive to the effects of excess adiposity than those with the II (or AA) genotype. Compared to other genotype carriers, participants with the ACE deletion genotype had a more significant impact of increasing childhood adiposity (respectively, BMI and triceps skin-fold thickness, but not including WC) on elevating adult DBP (limited support for SBP), and an elevated risk of hypertension. Notably, it may be possible to use ACE genotype to predict blood pressure response to childhood obesity. The worldwide obesity epidemic poses an enormous public health burden. As results may vary based on the target studied population, further research is required to determine the consistency in results as this study may inspire essential factors to consider for reducing hypertension among children and adults.

Limitations

The following factors may have limited this study to an extent: because we rely on two databases, PubMed and PubMed Central, the sample size of our analysis is quite limited, which may interfere with the actual quality of the primary research involved. Like any other possible problem in a systematic review, literature search bias occurs when relevant literature is not identified throughout the search process. However, we conducted a thorough search based on pre-defined parameters. Thus we can expect any such bias to be minimal. The variability of the inclusion criteria reduced the possibility of identifying papers relevant to our topic's breadth. Patients from various geographical regions and those aged 30 and under were included in the figures. As a result, the findings could be limited to specific populations.

## Conclusions

In conclusion, the results of this study support that childhood obesity may be a risk factor for several adult cardiovascular health risk factors. Cardiometabolic risk factors can accelerate the atherosclerotic process and heighten the risk of cardiovascular events, particularly when preventive and intervention measures are suboptimal. Several longitudinal and cohort studies have highlighted consistent findings between lifestyle habits like excessive nutrition or poor physical activity, and anthropometric parameters like increasing BMI, SBP, DBP, WC, high TG, high LDL, low HDL, pulse pressure, and glucose. The results were all strongly associated with the increased risk of cardiovascular disease. This review also included exploration of genetic risk factors between galectin-3, FTO, and ACE gene polymorphisms. Galactin-3 was strongly associated with cardiovascular risk factor outcomes bridged from childhood obesity. The FTO gene was also considerably more expressed in obese children and patients with established insulin resistance and was linked to an increase in WC, body fat, and a higher fasting free fatty acid concentration. Finally, although in the preliminary stages of the study, ACE deletion genotype was more significantly correlated to an increase in childhood adiposity, elevated adult diastolic blood pressure, and a higher risk of hypertension. The findings suggest that it may be possible to use the ACE genotype to predict blood pressure response to childhood obesity from an early age. These findings lend support to ways of lowering BMI at a young age, with the hopes of delaying the establishment of any precursors which may bring long-term and unfavorable cardiovascular outcomes. As a result, increasing our attention to the risk factors related to childhood hypertension could be beneficial for the primary prevention of its complications in adulthood.
